# A Cd(II) Luminescent Coordination Grid as a Multiresponsive Fluorescence Sensor for Cr(VI) Oxyanions and Cr(III), Fe(III), and Al(III) in Aqueous Medium

**DOI:** 10.3390/molecules26237103

**Published:** 2021-11-24

**Authors:** Kuo-Shun Liao, Meng-Jung Tsai, Li-Jen Hsu, Chih-Min Wang, Jing-Yun Wu

**Affiliations:** 1 Department of Applied Chemistry, National Chi Nan University, Nantou 545, Taiwan; aasd6307@gmail.com (K.-S.L.); s97324905@mail1.ncnu.edu.tw (M.-J.T.); Dodoadam1996@gmail.com (L.-J.H.); 2 Department of Bioscience and Biotechnology, National Taiwan Ocean University, Keelung 202, Taiwan; 3 General Education Center, National Taiwan Ocean University, Keelung 202, Taiwan

**Keywords:** coordination polymer, Cr(VI) oxyanion, luminescence sensor, trivalent metal ion

## Abstract

Hydro(solvo)thermal reactions of Cd(NO_3_)_2_, *N*-(pyridin-3-ylmethyl)-4-(pyridin-4-yl)-1,8-naphthalimide (NI-mbpy-34), and 5-bromobenzene-1,3-dicarboxylic acid (Br-1,3-H_2_bdc) afforded a luminescent coordination polymer, {[Cd(Br-1,3-bdc)(NI-mbpy-34)(H_2_O)]∙2H_2_O}*_n_* (**1**). Single-crystal X-ray diffraction analysis showed that **1** features a two-dimensional (2-D) gridlike **sql** layer with the point symbol of (4^4^·6^2^), where the Cd(II) center adopts a {CdO_5_N_2_} pentagonal bipyramidal geometry. Thermogravimetric (TG) analysis confirmed the thermal stability of **1** up to about 340 °C, whereas XRPD patterns proved the maintenance of crystallinity and framework integrity of **1** in CH_2_Cl_2_, H_2_O, CH_3_OH, and toluene. Photoluminescence studies indicated that **1** displayed intense blue fluorescence emissions in both solid-state and H_2_O suspension-phase. Owing to the good fluorescent properties, **1** could serve as an excellent turn-off fluorescence sensor for selective and sensitive Cr(VI) detection in water, with LOD = 15.15 μM for CrO_4_^2^^−^ and 14.91 μM for Cr_2_O_7_^2^^−^, through energy competition absorption mechanism. In addition, **1** could also sensitively detect Cr^3+^, Fe^3+^, and Al^3+^ ions in aqueous medium via fluorescence-enhancement responses, with LOD = 2.81 μM for Cr^3+^, 3.82 μM for Fe^3+^, and 3.37 μM for Al^3+^, mainly through an absorbance-caused enhancement (ACE) mechanism.

## 1. Introduction

With the advanced development of modern society, rapid industrial and agricultural productions and rich human activities have increasingly brought about severe chemical pollution. Among various chemical pollutants, heavy metal ion based inorganic contaminates are of higher concern compared with other contaminants such as organic pollutants due to their nondegradability and bioaccumulation [1]. Moreover, heavy metal ions are well-known poisonous contaminants in water due to their high toxicity, which could cause serious environmental and ecological harm and cause a detrimental effect on human health [2]. For example, chromium exists in aquatic environments usually in the forms of Cr(VI) oxyanions, i.e., dichromate (Cr_2_O_7_^2^^−^) and chromate (CrO_4_^2^^−^) ions, and/or free cation, i.e., trivalent Cr(III) ion. The Cr(VI) oxyanions are highly carcinogenic and mutagenic, causing hereditary genetic defects and various types of cancers [3,4,5]. While the Cr(III) ion is essentially harmless due to its low toxicity, it may, however, cause mutations and malignant cells when excessive accumulation occurs [5,6,7]. The permissible limit for Cr(VI) in drinking water is set as 50 μg/L by the World Health Organization (WHO) [8]. Iron and aluminum are two ubiquitous metals widely used in daily applications around human living environments [9]. Trivalent Fe(III) and Al(III) cations are the forms of iron and aluminum that can enter human body. As one of the most important elements for living organisms, Fe(III) ion influences a variety of vital bioprocesses such as electron transfer, oxygen storage, oxygen metabolism, among others [10,11,12]. The deficiency or excess of Fe(III) ion is harmful to human health, resulting in some diseases [13,14,15]. Additionally, Al(III) ion in body fluids is toxic to humans and will induce harmful effects that cause diseases such as Alzheimer’s disease and Parkinson’s disease when its content in the body is over an acceptable standard [9,12,16]. The tolerable daily ingestion of Al^3+^ for the human body is about 3–10 mg/day [7] and the permissible level in drinking water is set as 7.41 μM by the WHO [17].

In recent years, the sensing and detection of chemical pollutants has attracted tremendous interest from many researchers. Benefitting from their unique merits, low-cost, easy manipulation, instant, visual identification, excellent sensitivity, and high selectivity [7,12], fluorescence based detection has gained considerable attention among various conventional instrumental techniques. Nowadays, various advanced fluorescence sensory materials have emerged, such as organic dyes [18], quantum dots (QDs) [19,20], and carbon dots (CDs) [21,22], among others. Of particular note is one kind of organic–inorganic hybrid material called coordination polymers (CPs), fabricated by metal ions or clusters as nodes and bridging organic ligands as linkers through the connection of coordination bonds. As a matter of fact, luminescent CP-based chemo/biosensors have been actively used to detect small molecules, explosives, ions, gas, and pH, among others, and several reviews have been devoted to the sensory properties [23,24,25,26,27,28,29]. Recently, we have made advances in fluorescence detection of hazardous chemical contaminants by using luminescent organic–inorganic hybrid materials as sensory platforms [30,31,32,33,34,35,36,37,38,39]. Herein, we report a new Cd(II) based luminescent CP, {[Cd(Br-1,3-bdc)(NI-mbpy-34)(H_2_O)]∙2H_2_O}*_n_* (**1**), where NI-mbpy-34 = *N*-(pyridin-3-ylmethyl)-4-(pyridin-4-yl)-1,8-naphthalimide, Br-1,3-H_2_bdc = 5-bromobenzene-1,3-dicarboxylic acid. The solid-state structure of **1** has a simple two-dimensional (2-D) layer structure adopting a gridlike net with the point symbol of (4^4^·6^2^), which is highly stable in water. The remarkable emission properties make **1** a functional multiresponsive fluorescence sensor for Cr(VI) oxyanions detection via a quenching effect, and Cr(III), Fe(III), and Al(III) sensing via an enhancement response in aqueous medium, with high sensitivity and remarkable selectivity.

## 2. Experimental Section

### 2.1. Materials and Characterization

All of the chemicals and solvents were acquired from market sources (MATRIX, ULTRA, ACROS, PanReac Applichem, SHOWA, Fluka, VETEC, ALFA, MACRON, J.T. Baker, SIGMA ALDRICH), and used without further processing. Ligand NI-mbpy-34 was synthesized according to the previously reported literature [40]. Thermogravimetric (TG) analyses were performed using a Thermo Cahn VersaTherm HS TG analyzer (Thermo, Newington, NH, USA) under flow nitrogen with a heating rate of 5 °C/min. X-ray powder diffraction (XRPD) patterns were measured using a Shimadzu XRD-7000 diffractometer (Shimadzu, Kyoto, Japan) with a graphite monochromatized Cu Kα radiation (λ = 1.5406 Å) at 30 kV and 30 mA. Infrared (IR) spectroscopic measurements were performed on a Perkin-Elmer Frontier Fourier transform infrared spectrometer (Perkin-Elmer, Taipei, Taiwan) using attenuated total reflection (ATR) technique. Fluorescence spectroscopic measurements were performed at room temperature using a Hitachi F7000 fluorescence spectrophotometer (Hitachi, Tokyo, Japan) equipped with a 150 W xenon lamp as an excitation source. UV-Vis absorption spectra were recorded at room temperature using a JASCO V-750 UV/VIS spectrophotometer (JASCO, Tokyo, Japan). Elemental microanalyses (C, H, N) were performed on an Elementar Vario EL III analytical instrument (Elementar, Langenselbold, Germany). Ultrasonic agitation of suspensions was conducted using a Qsonica Q125 instrument. X-ray photoelectron spectroscopy (XPS) analyses were performed on an ULVAC-PHI PHI 5000 VersaProbe/Scanning ESCA Microprobe instrument (ULVACPHI Inc., Kanagawa, Japan).

### 2.2. Synthesis of {[Cd(Br-1,3-bdc)(NI-mbpy-34)(H_2_O)]∙2H_2_O}_n_ (**1**)

A DMF solution (2 mL) of NI-mbpy-34 (9.1 mg, 0.025 mmol), an aqueous solution (2 mL) of Cd(NO_3_)_2_∙4H_2_O (15.4 mg, 0.050 mmol), and a DMF solution of Br-1,3-H_2_bdc (12.3 mg, 0.050 mmol) were sequentially added into an acid digestion bomb placed in a Teflon-lined stainless steel autoclave. The mixture was kept inside a furnace at 80 °C for 48 h and then cooled to ambient temperature. Yellowish crystals suitable for X-ray diffraction were collected after washing with distilled water and ethanol, and dried at room temperature. Yield: 60% based on NI-mbpy-34 (12.1 mg, 0.015 mmol). IR (ATR, cm^−1^): 3072, 1590, 1460, 1333, 992, 889, 723. Anal. Calcd. for C_31_H_24_BrCdN_3_O_9_: C, 48.01; H, 3.09; N, 5.42%. Found: C, 48.24; H, 2.94; N, 5.41%. The phase purity of the bulky sample was confirmed by the closely matched XRPD patterns between the simulated pattern, calculated from single-crystal diffraction data and the experimental pattern of as-synthesized **1** without grinding (Figure S1). Of particular note, the XRPD patterns of the same microcrystalline sample after grinding showed alternations in intensity, and in some peak positions, compared to the simulated XRPD patterns. This is tentatively attributed to the influences of either the variation in preferred orientation of the powdered sample [30] or the partial crystal structure distortion caused by grinding [41] or both.

### 2.3. Single-Crystal X-ray Structure Determinations

The diffraction data were collected using a Bruker D8 Venture diffractometer configured with a PHOTO100 CMOS detector at 150(2) K, equipped with a graphite monochromated Mo Kα radiation (λ = 0.71073 Å). The structures were solved by direct methods with the SHELXTL program [42] and refined by full-matrix least-squares methods on *F*^2^ using the SHELXL-2014/7 [43], incorporated in WINGX [44] crystallographic collective package. Non-hydrogen atoms were refined with anisotropic displacement parameters, except where noted. Carbon-bound hydrogen atoms were calculated in ideal positions and refined as riding mode. Oxygen-bound hydrogen atoms were structurally evident in the difference Fourier map. All of the hydrogen atoms were refined with isotropic displacement parameters, *U_iso_*, constrained to be 1.2 or 1.5 times *U_eq_* of the carrier atom. Experimental details for X-ray data collection and the refinements are summarized in Table 1. Hydrogen-bonding parameters are shown in Table S1. CCDC 1991627 (**1**) contain the supplementary crystallographic data for this paper. These data can be obtained free of charge from the Cambridge Crystallographic Data Centre via www.ccdc.cam.ac.uk/data_request/cif (accessed on 19 March 2020).

## 3. Results and Discussion

### 3.1. Synthsis and Crystal Structure of {[Cd(Br-1,3-bdc)(NI-mbpy-34)(H_2_O)]∙2H_2_O}_n_ (**1**)

**Scheme 1:**
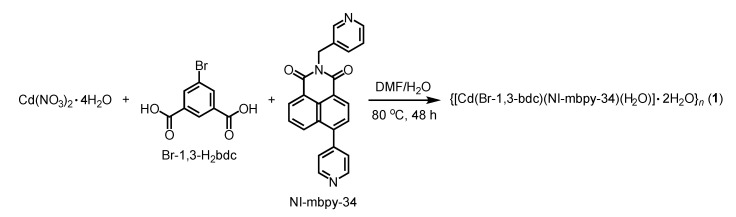
Synthesis of **1**.

**Figure 1:**
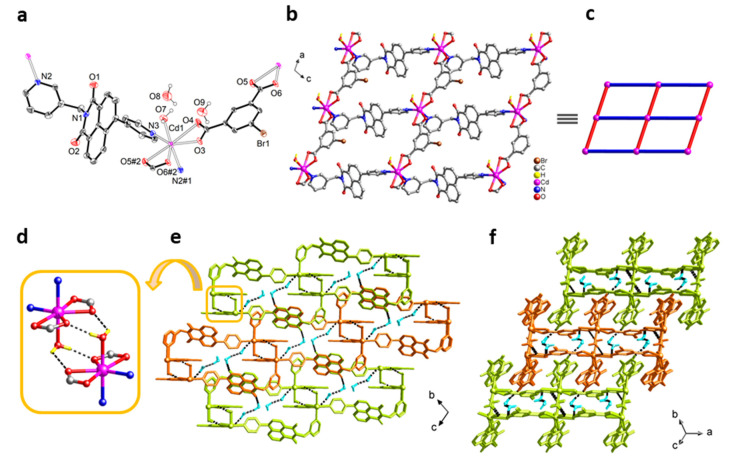
Crystal structure of **1**: (**a**) ORTEP plot of the asymmetry unit with thermal ellipsoids in 50% probability level, showing the coordination environment around the Cd(II) center and the coordination modes of the Br-1,3-bdc^2−^ and NI-mbpy-34 ligands. Symmetry codes: #1, *x* + 1, *y* + 1, *z* + 1; #2, *x* − 1, *y*, *z*; (**b**) a single 2-D gridlike layer; (**c**) schematic representation of the 4-connected **sql** grid; (**d**) highlight of the hydrogen bonding interactions between the coordinated water molecules and the carboxylate oxygen atoms of the Br-1,3-bdc^2−^ ligands; (**e**) representation of the 2-D hydrogen-bonded bilayers viewed down the crystallographic [100] direction, showing small pores accompanied by lattice water molecules; (**f**) representation of the lattice water molecules supported 3-D hydrogen-bonded network viewed down the [111] direction, showing the intercalated 2-D hydrogen-bonded bilayers.

Hydro(solvo) thermal reactions of Cd(NO_3_)_2_∙4H_2_O, Br-1,3-H_2_bdc, and NI-mbpy-34 in DMF/H_2_O media afforded **1** as yellowish crystals (Scheme 1). Single-crystal X-ray structure analysis reveals that the crystal structure of **1** belongs to the triclinic space group *P*$\bar{1}$. The asymmetric unit contains one Cd(II) center, one Br-1,3-bdc^2−^ dianion, one NI-mbpy-34 ligand, and one coordination and two lattice water molecules (Figure 1a). The Cd(II) center adopts a {CdO_5_N_2_} pentagonal bipyramidal geometry, where the equatorial plane is made up of two carboxylate groups of two distinct Br-1,3-bdc^2−^ dianions in one asymmetric (Cd1–O3 = 2.254(2) Å, Cd1–O4 = 2.753 Å) and one symmetric (Cd1–O5#2 = 2.393(2) Å, Cd1–O6#2 = 2.389(2) Å, #2, *x* − 1, *y*, *z*) chelating modes and one 4-pyridyl nitrogen atom (naphthalene end) from one NI-mbpy-34 ligand (Cd1–N3 = 2.301(2) Å), while the two apical positions are located by one coordination water molecule (Cd1–O7 = 2.348(2) Å) and one 3-pyridyl nitrogen atom (imide end) from the other NI-mbpy-34 ligand (Cd1–N2#1 = 2.354(3) Å, #1, *x* + 1, *y* + 1, *z* + 1). Each Br-1,3-bdc^2−^ dianion adopts a μ_2_-Br-1,3-bdc-κO,Oʹ:κO,Oʹ mode to bridge two Cd(II) centers, where the two carboxylate groups suit an asymmetric and a symmetric chelating coordination mode (Figure 1a). Connection of Cd(II) centers by Br-1,3-bdc^2−^ dianions and NI-mbpy-34 ligands simultaneously forms a two-dimensional (2-D) gridlike layer (Figure 1b), which can be simplified as a 4-connected **sql** net with the point symbol of (4^4^·6^2^) (Figure 1c). Two such gridlike layers with the coordination water molecules oriented face-to-face are linked together in pair through O–H∙∙∙O hydrogen bonding interactions (O∙∙∙O, 2.723(3) and 2.932(3) Å, Table S1) formed between the coordinated water molecules and the carboxylate oxygen atoms of the Br-1,3-bdc^2^^−^ ligands (Figure 1d), generating a 2-D hydrogen-bonded bilayer (Figure 1e). When viewed down the crystallographic [100] direction, there are small pores with potential sufficient solvent accessible voids of only about 9.8% of the unit cell volume [45], accompanied by lattice water molecules (Figure 1e). These lattice water molecules form hydrogen-bonding interactions with each other (O∙∙∙O, 3.035(5) Å) and, importantly, with the framework (O∙∙∙O, 2.839(5) and 2.855(4) Å) to expand the 2-D hydrogen-bonded bilayers to be the three-dimensional (3-D) hydrogen-bonded network (Figure 1e,f).

### 3.2. Chemical Stability and Thermal Properties

**Figure 2:**
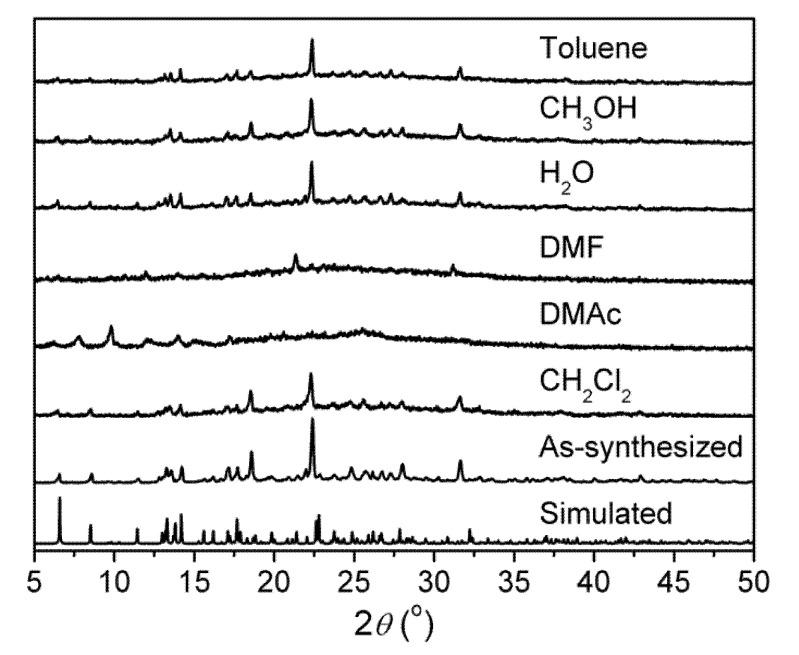
XRPD patterns of **1** before and after dispersing in different solvents for 24 h.

The chemical stability of **1** in different solvents including dichloromethane (CH_2_Cl_2_), *N*,*N*′-dimethylacetamide (DMAc), *N*,*N*′-dimethylformamide (DMF), H_2_O, methanol (CH_3_OH), and toluene was checked. After separately immersing **1** in CH_2_Cl_2_, H_2_O, CH_3_OH, and toluene for 24 h, the checked XRPD patterns of so-obtained powdered samples were very similar to the patterns of as-synthesized **1** after grinding, with slight differences in peak intensity (Figure 2). This might suggest the preferred orientation effect or the minor extent of partial distortion of the long range order in **1**. However, the checked XRPD patterns still imply the maintenance of framework integrity and crystallinity, confirming the high stability of **1**. In contrast, **1** displayed low stability after immersing in DMAc and DMF due to the poorly matched XRPD profiles.

Thermogravimetric (TG) analysis was performed under a nitrogen atmosphere to examine the thermal stability of **1** (Figure S2). The TG trace of **1** exhibited a weight loss of 5.4% from room temperature to 69 °C, corresponding to the escape of lattice water molecules (calcd. 4.6%). A gradual weight loss of 1.6% corresponding to the removal of coordinated water molecules (calcd. 2.3%) followed when the temperature was raised to approaching 186 °C. Then, the TG trace showed the existence of a stable plateau before the framework began to process a two-step collapse from ca. 340 °C to ca. 633 °C. During the decomposition, bromide might react with divalent cadmium to generate CdBr_2_ (b.p. = 844 °C), which would escape at higher temperature. The final residue of 8.4% was reasonably assigned to the CdO component (calcd. 8.3%).

### 3.3. Gas Adsorption Properties of Activated **1**

In the crystal structure of **1**, there are free void spaces of about 9.8% of the unit cell volume, hence, the porous properties of activated **1** were investigated by gas adsorption studies. Prior to gas adsorption experiments, as-synthesized **1** (about 100 mg) was thermally treated at 100 °C under a reduced pressure for 24 h to remove solvent molecules and thus to give activated **1**. For activated **1**, N_2_ adsorption isotherms exhibited no appreciable uptakes of 5.92 cm^3^ g^−1^ STP at *P*/*P*_0_ = 1 and 77 K, whereas CO_2_ adsorption isotherms showed negligible uptakes of 11.19 cm^3^ g^−1^ STP at *P*/*P*_0_ = 1 and 195 K (Figure S3). The low N_2_ and CO_2_ uptakes of thermally activated **1** might be attributed to the small sufficient solvent-accessible voids and framework distortion induced pore-reduction, which resulted in surface adsorption. The latter assumption was supported by the checked XRPD patterns, which showed obvious differences with the experimental profiles of as-synthesized sample of **1** (Figure S4).

### 3.4. Photoluminescence Properties

When excited at *λ*_ex_ = 370 nm, the solid-state emission spectrum of NI-mbpy-34 showed an intense emission band centered at 462 nm, which was overlapped with two further bands as shoulders at around 433 and 480 nm (Figure S5). After irradiation at *λ*_ex_ = 360 nm, Br-1,3-H_2_bdc emitted an extremely weak solid-state emission centered at around 468 nm. Comparably, **1** emitted intense blue fluorescence with an emission band centered at 436 nm in solid-state and 422 nm in H_2_O suspension-phase upon excitation at *λ*_ex_ = 365 nm. From the band position and shape, the emissions were tentatively assigned to the intraligand charge transfer of the NI-mbpy-34 ligand perturbed by metal coordination; this is further supported by the high resemblance in excitation spectra between NI-mbpy-34 and **1**.

### 3.5. Detection of Anions

**Figure 3:**
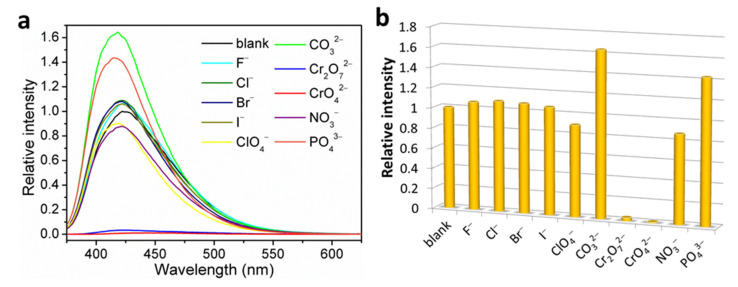
(**a**) Fluorescence spectra and (**b**) bar diagrams of **1** in H_2_O suspension-phase upon addition of different anions at 1.0 mM when excited at *λ*_ex_ = 365 nm.

**Figure 4:**
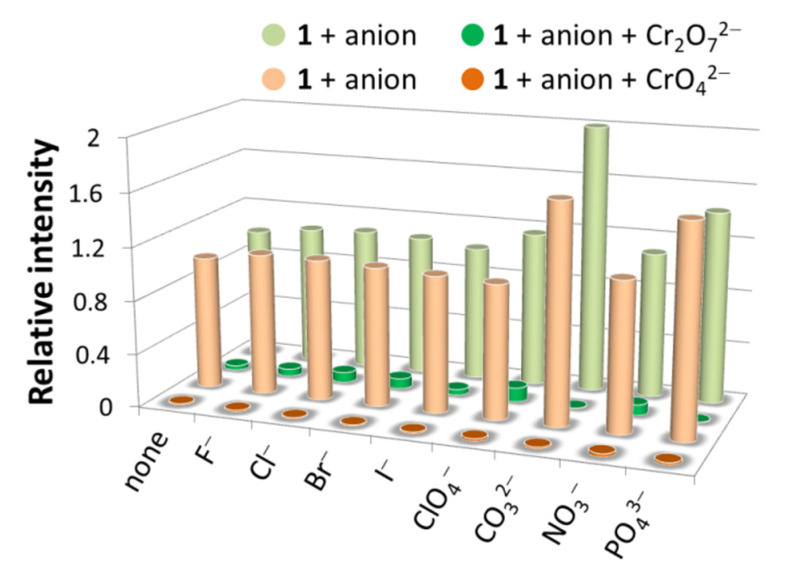
Bar diagrams to show luminescence quenching of **1** in H_2_O suspension-phase for Cr_2_O_7_^2^^−^ and CrO_4_^2^^−^ ions with different perturbed anions in equal concentration (1.0 mM).

In view of the high water stability and excellent fluorescence properties of **1** in H_2_O suspension-phase, its potential ability to detect anions was explored in water. Anion detection studies were carried out by separately adding aqueous solutions of NaF and K*_m_*X (X*^m^*^−^ = Cl^−^, Br^−^, I^−^, ClO_4_^−^, CO_3_^2−^, Cr_2_O_7_^2^^−^, CrO_4_^2^^−^, NO_3_^−^, and PO_4_^3−^, *m* = 1, 2, 3) into the well-prepared H_2_O suspensions of **1**, with a concentration of 1 mM. Upon excitation at *λ*_ex_ = 365 nm, the fluorescence detection results showed that most anions had an inconspicuous fluorescence intensity change effect (<10% change) on **1**, except for the CO_3_^2−^, Cr_2_O_7_^2^^−^, CrO_4_^2^^−^, and PO_4_^3−^ ions (Figure 3). It is noted that CO_3_^2−^ and PO_4_^3−^ caused significant fluorescence enhancement by 63% and 44%, respectively. However, the two Cr(VI) oxyanions, Cr_2_O_7_^2^^−^ and CrO_4_^2^^−^, showed obviously high quenching effects with an efficiency up to 97% and 99%, respectively, (quenching efficiency (%) = (*I*_0_ − *I*)/*I*_0_ × 100%, where *I*_0_ and *I* are the maximum fluorescence intensities of **1** before and after addition of a quencher). Therefore, it is suggested to use the fluorescence quenching of **1** to detect trace amounts of Cr_2_O_7_^2^^−^ and CrO_4_^2^^−^ ions in water media. The anti-interference ability of **1** toward Cr_2_O_7_^2^^−^ and CrO_4_^2^^−^ was examined with the existence of different competitive anions in equal concentration. The competition experiments clearly indicated that Cr_2_O_7_^2^^−^ and CrO_4_^2^^−^ both retained high quenching ability to almost completely turn off the fluorescence of **1** when the other interference anions are present (Figure 4), implying high selectivity of **1** toward Cr_2_O_7_^2^^−^ and CrO_4_^2^^−^ over others perturbed anions in water media.

**Figure 5:**
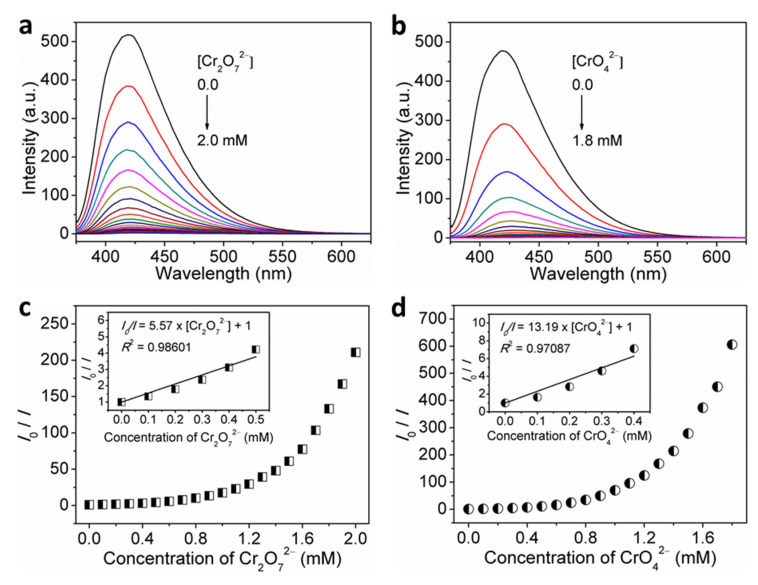
(**a**,**b**) Concentration-dependent fluorescence emission spectra of **1** in H_2_O suspension-phase by incremental addition of Cr_2_O_7_^2^^−^ and CrO_4_^2^^−^ upon excitation at *λ*_ex_ = 365 nm. (**c**,**d**) Stern−Volmer plot over the titration concentrations of Cr_2_O_7_^2^^−^ and CrO_4_^2^^−^ for **1** in H_2_O suspension-phase. Inset: the linear Stern−Volmer plot at low Cr_2_O_7_^2^^−^ and CrO_4_^2^^−^ ion concentrations, where the solid line represents the linear fit to the Stern−Volmer equation.

The sensitivity of Cr(VI) oxyanions detection in water media can be evaluated by quantitative analysis and limit of detection (LOD) values. The concentration-dependent fluorescence intensity of **1** was determined by gradually adding different concentrations of Cr(VI) oxyanion into well-dispersed H_2_O suspensions of **1**. As observations, the fluorescence intensity of **1** gradually decreased with increasing concentration of Cr_2_O_7_^2^^−^ and CrO_4_^2^^−^ (Figure 5a,b), indicating that the fluorescence quenching of **1** caused by the introduction of Cr_2_O_7_^2^^−^ and CrO_4_^2^^−^ ions can be quantified. It is also noted that the fluorescence intensity (*λ*_em_ = 414 nm) versus the concentration of Cr_2_O_7_^2^^−^ and CrO_4_^2^^−^ can be well fitted to the first-order exponential decay (Figure S6), suggesting a diffusion-controlled fluorescence quenching. The fluorescence quenching efficiencies were further analyzed by a Stern−Volmer analysis based on the equation: *I*_0_/*I* = 1 + *K*_SV_[Q], where *I*_0_ and *I* are the fluorescence intensities before and after the addition of analytes, respectively, [Q] is the molar concentration of the analyte (mM), and *K*_SV_ is the Stern−Volmer quenching constant (M^−1^). The Stern–Volmer plots exhibited good linear correlations at low concentrations (inset in Figure 5c,d), which gave the *K*_SV_ values of 5.56 × 10^3^ M^−1^ for Cr_2_O_7_^2^^−^ and 1.32 × 10^4^ M^−1^ for CrO_4_^2^^−^. Furthermore, the Stern–Volmer plots displayed upward deviation from linearity at high concentrations (Figure 5c,d), as a result of the combination of a dynamic energy transfer mechanism and a static self-absorption mechanism [46,47]. The LOD values for Cr_2_O_7_^2^^−^ and CrO_4_^2^^−^ were determined using the equation: LOD = 3*σ*/*k*, where *σ* is the standard deviation of five blank measurements of fluorescence for the H_2_O suspensions of **1** and *k* is the absolute value of the slope of the calibration curve at low concentrations. The LOD values for Cr_2_O_7_^2^^−^ and CrO_4_^2^^−^ were determined to be 14.91 (3.22) and 15.15 (1.76) μM (ppm), respectively, (Figure S7). Owing to the excellent anti-interference ability and high *K*_SV_ and lower LOD values, **1** can be an effective fluorescence sensor displaying high detection selectivity and sensitivity toward Cr_2_O_7_^2^^−^ and CrO_4_^2^^−^ in water media.

### 3.6. Detection of Metal Ions

**Figure 6:**
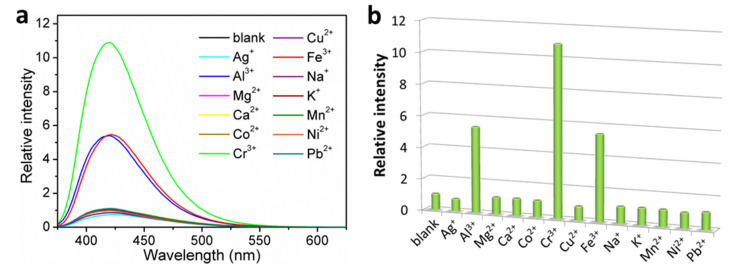
(**a**) Fluorescence spectra and (**b**) bar diagrams of **1** in H_2_O suspension-phase upon addition of different metal ions at 1.0 mM when excited at *λ*_ex_ = 365 nm.

**Figure 7:**
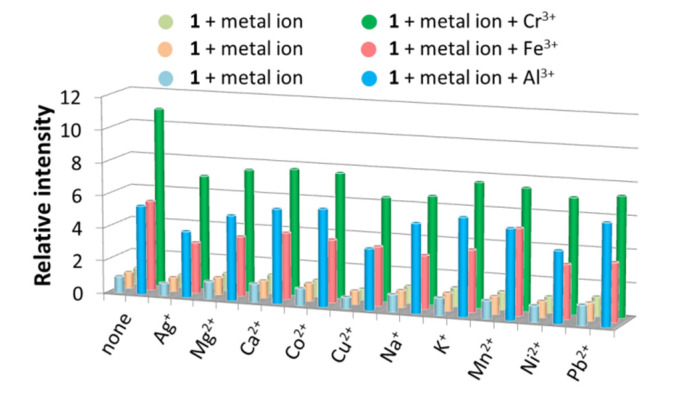
Bar diagrams to show fluorescence enhancement of **1** in H_2_O suspension-phase for Cr^3+^, Fe^3+^, and Al^3+^ ions with different competitive metal ions in equal concentration (1.0 mM).

The influence of different metal ions on the fluorescent properties of **1** in H_2_O suspension-phase was also investigated through similar procedures by adding well-prepared aqueous solutions of metal ions, including M(NO_3_)*_n_* (M*^n^*^+^ = Ag^+^, Al^3+^, Mg^2+^, Ca^2+^, Co^2+^, Cr^3+^, Cu^2+^, Fe^3+^, Na^+^, K^+^, Mn^2+^, Ni^2+^, and Pb^2+^, *n* = 1, 2, 3), into the H_2_O suspension of **1**, with a concentration of 1 mM. Upon excitation at *λ*_ex_ = 365 nm, interestingly, trivalent metal ions of Cr^3+^, Fe^3+^, and Al^3+^ have striking enhancement responses of 10.9, 5.4, and 5.4 times on the fluorescence intensity of **1** in H_2_O suspension-phase, while other mono- and divalent metal ions have no or only minor effects (intensity change ≤ 10%) on the fluorescence intensity (Figure 6). It is also noteworthy that the addition of metal ions did not cause significant wavelength shift. To examine the selectivity of **1** toward Cr^3+^, Fe^3+^, and Al^3+^, the interference experiments were carried out. After adding Cr^3+^, Fe^3+^, and Al^3+^ ions into the H_2_O suspension of **1** containing other competitive metal ions in equal concentration, the fluorescence intensity of **1** in H_2_O suspension-phase increased immediately but showed a certain degree of reduction in some cases, with a maximum of about 43% reduction compared to that without the co-existence of competitive metal ions (Figure 7). The observations indicated that changes in intensity for sensing Cr^3+^, Fe^3+^, and Al^3+^ were somewhat negatively affected, but still within acceptable limits, by the competitive metal ions. As a result, **1** displayed accessible anti-interference ability and detection selectivity for recognizing Cr^3+^, Fe^3+^, and Al^3+^ over other competitive metal ions.

**Figure 8:**
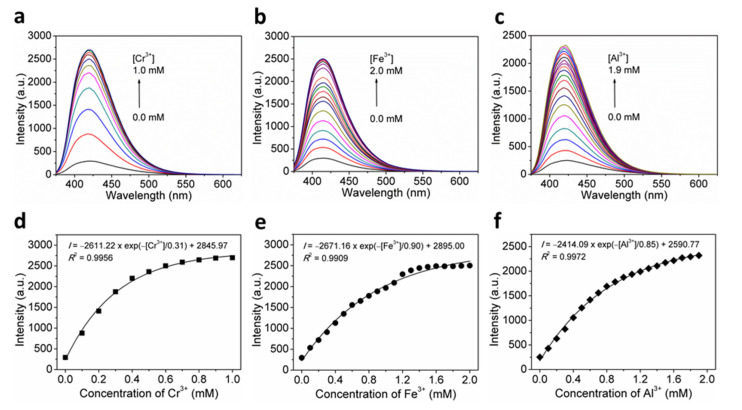
(**a**–**c**) Concentration-dependent fluorescence emission spectra of **1** in H_2_O suspension-phase by incremental addition of Cr^3+^, Fe^3+^, and Al^3+^ ions upon excitation at *λ*_ex_ = 365 nm. (**d**–**f**) Plot of fluorescence intensity at *λ*_em_ = 414 nm versus Cr^3+^, Fe^3+^, or Al^3+^ ion concentration for **1** in H_2_O suspension-phase, showing the first-order exponential decay.

To further quantitatively evaluate the fluorescence response of **1** in H_2_O suspension-phase toward Cr^3+^, Fe^3+^, and Al^3+^, fluorescence titration for the above three metal ions was also investigated. It was observed that the fluorescence intensity of **1** in H_2_O suspension-phase increased gradually upon incremental addition of the aqueous solutions of Cr^3+^, Fe^3+^, and Al^3+^ ions (Figure 8a–c). The fluorescence intensity (*λ*_em_ = 414 nm) obeyed the first-order exponential decay relationship with the concentration of Cr^3+^, Fe^3+^, and Al^3+^ (Figure 8d–f), suggesting diffusion-controlled fluorescence enhancement. The curvilinear dependence between *I* and concentration of metal ion conforms to the equation *I* = −2611.22 × exp(−[Cr^3+^]/0.31) + 2845.97 (*R*^2^ = 0.9956) for Cr^3+^ detection, *I* = −2671.16 × exp(−[Fe^3+^]/0.90) + 2895.00 (*R*^2^ = 0.9909) for Fe^3+^ detection, and *I* = −2414.09 × exp(−[Al^3+^]/0.85) + 2590.77 (*R*^2^ = 0.9972) for Al^3+^ detection. Moreover, there is a linear relationship between the fluorescence intensity of **1** in H_2_O suspension-phase and the Cr^3+^ concentration in the range of 0−0.4 mM, the Fe^3+^ concentration in the range of 0−0.6 mM, and the Al^3+^ concentration in the range of 0−0.7 mM (Figure S8), suggesting that **1** could potentially quantitatively determine Cr^3+^, Fe^3+^, and Al^3+^ ions. Accordingly, the LOD is calculated to be 2.81 (146.2) μM (ppb) for Cr^3+^ detection, 3.82 (198.7) μM (ppb) for Fe^3+^ detection, and 3.37 (90.9) μM (ppb) for Al^3+^ detection, through the equation of 3*σ*/*k*.

### 3.7. Fluorescence-Responsive Sensing Mechanisms

Herein, the possible fluorescence-responsive sensing mechanisms are elucidated. For Cr(VI) oxyanions detection, the XRPD patterns of **1** treated with Cr_2_O_7_^2^^−^ and CrO_4_^2^^−^ in water were closely matched to that of as-synthesized **1** (Figure S9), implying that the framework integrity of **1** was retained. Thus, the turn-off sensing mechanism could not be due to the collapse of the framework. Ultimately, the mechanism of the quenching effect could be interpreted by UV−vis absorption spectroscopy. The UV−vis absorption band of Cr(VI) oxyanions and the fluorescence emission band of **1** show a small degree of overlap, suggesting that fluorescence quenching was not caused by energy transfer mechanism (Figure S10). However, the large extent of overlap between the UV−vis absorption spectra of Cr_2_O_7_^2^^−^/CrO_4_^2^^−^ and the fluorescence excitation spectrum of **1** demonstrates that the competition of absorption of irradiated light source energy between the Cr_2_O_7_^2^^−^ and CrO_4_^2^^−^ ions and **1** leads to the high fluorescence quenching efficiencies.

The XRPD patterns of **1** after being treated with Cr^3+^, Fe^3+^, and Al^3+^ in water were very similar to that of as-synthesized **1** (Figure S11), suggesting that **1** kept the framework integrity and crystallinity. This excluded the possibility of framework collapse caused fluorescence enhancement. Literature has shown that framework–analyte interactions usually demonstrated one of the most possible mechanisms for fluorescence turn-on detection toward metal ions [12,13,31,40,48,49]. To verify this assumption, IR and X-ray photoelectron spectroscopy (XPS) spectra were measured. Unfortunately, the IR spectra of **1** did not show significant changes after immersion (Figure S12). Meanwhile, the O 1s and N 1s peaks in the XPS spectra were almost unchanged (Figure S13). These findings indicate that there might be no framework–analyte interactions or that such interactions are too weak to cause spectra change. Thus, the turn-on detection mechanism is not due to the framework–analyte interactions. Interestingly, the UV–vis absorption spectra of **1** after addition of Cr^3+^, Fe^3+^, and Al^3+^ exhibited significant increase in the absorbance within the sphere of 300–400 nm (Figure S14), corresponding to the excitation energy used. The results illustrated that the turn-on detection of **1** toward Cr^3+^, Fe^3+^, and Al^3+^ can be explained by the absorbance caused enhancement (ACE) mechanism [12,50], meaning that the M^3+^-treated **1** would release more energy than pristine **1** to demonstrate turn-on effect in the emission process.

## 4. Conclusions

In summary, a luminescent Cd(II) coordination polymer **1** adopting a 2-D gridlike **sql** layer has been successfully synthesized. Coordination polymer **1** shows strong fluorescence emissions in solvent suspension-phase, making them potential candidates to be employed in sensing Cr_2_O_7_^2−^ and CrO_4_^2−^ ions via fluorescence quenching effect and in detecting Cr^3+^, Fe^3+^, and Al^3+^ ions via fluorescence enhancement response, with high sensitivity and selectivity. From the fluorescence-sensing mechanism studies, absorption energy competition and absorbance caused enhancement can, respectively, interpret the quenching effect toward Cr(VI) oxyanions and the enhancement effect toward Cr^3+^, Fe^3+^, and Al^3+^ metal ions.

## Figures and Tables

**Table 1:** X-ray data collection and structure refinement for **1**.

Empirical Formula	C_31_H_24_BrCdN_3_O_9_
*M*_w_	774.84
Crystal system	Triclinic
Space group	*P* $\bar{1}$
*a*, Å	10.169(3)
*b*, Å	11.285(4)
*c*, Å	14.502(4)
*α*, °	97.526(10)
*β*, °	107.465(10)
*γ*, °	108.503(10)
*V*, Å^3^	1457.8(8)
*Z*	2
*T*, K	150(2)
λ, Å	0.71073
*D_calc_*, g cm^−3^	1.765
*F*_000_	772
*μ*, mm^−1^	2.182
Reflns collected	44,573
Unique reflns (*R*_int_)	5934(0.0424)
Obsd reflns (*I* > 2*σ* (*I*))	5201
Params	406
*R*_1_*^a^* (*I* > 2*σ* (*I*))	0.0289
*wR*_2_*^b^* (*I* > 2*σ* (*I*))	0.0601
*R*_1_*^a^* (all data)	0.0368
*wR*_2_*^b^* (all data)	0.0646
GOF on *F*^2^	1.152
Δρ_max_, Δρ_min_, e Å^−3^	1.015, −0.561

${}^{a}\;R_{1}=\sum \left|\left|F_{o}\right|-\left|F_{c}\right|\right|/\sum \left|F_{o}\right|$; ${}^{b}\;wR_{2}=\{\sum \left[w{\left(F_{o}{}^{2}-F_{c}{}^{2}\right)}^{2}\right]/\sum \left[w{\left(F_{o}{}^{2}\right)}^{2}\right]{\}}^{1/2}$.

## Data Availability

Not available.

## Supplementary Materials

The following are available online, Figure S1. Experimental and simulated XRPD patterns for as-synthesized **1**. Table S1. Hydrogen-bonding parameters in **1**. Figure S2. TG curve of **1**. Figure S3. Gas adsorption isotherms for thermally activated **1** conducted at 77 K for N_2_ and 195 K for CO_2_. Figure S4. XRPD patterns of **1**: simulated, as-synthesized, and after N_2_ and CO_2_ adsorption. Figure S5. Fluorescence excitation and emission spectra of NI-mbpy-34, Br-1,3-H_2_bdc, and **1** in solid-state, as well as **1** in H_2_O suspension-phase at room temperature. Figure S6. Fluorescence intensity traces for the H_2_O suspensions of **1** upon incremental addition of Cr_2_O_7_^2^^−^ and CrO_4_^2^^−^ ions, following the first-order exponential decay. Conditions: *λ*_em_ = 414 nm (*λ*_ex_ = 365 nm). Figure S7. Linear region of fluorescence intensity for the H_2_O suspensions of **1** upon incremental addition of Cr_2_O_7_^2^^−^ and CrO_4_^2^^−^ ions. The following table lists the relevant parameters of LOD for the H_2_O suspensions of **1** toward Cr_2_O_7_^2^^−^ and CrO_4_^2^^−^ ions. Conditions: *λ*_em_ = 420 nm (*λ*_ex_ = 365 nm). Figure S8. Linear region of fluorescence intensity for the H_2_O suspensions of **1** upon incremental addition of Cr^3+^, Fe^3+^, and Al^3+^ ions. The following table lists the relevant parameters of LOD for the H_2_O suspensions of **1** toward Cr^3+^, Fe^3+^, and Al^3+^ ions. Conditions: *λ*_em_ = 414 nm (*λ*_ex_ = 365 nm). Figure S9. XRPD patterns of **1** before and after immersing in Cr_2_O_7_^2^^−^ and CrO_4_^2^^−^ aqueous solutions for 24 h. Figure S10. Spectral overlap between the normalized absorption spectra of Cr_2_O_7_^2^^−^ and CrO_4_^2^^−^ in aqueous solutions and the normalized excitation and emission spectra of **1** in H_2_O suspensions. Figure S11. XRPD patterns of **1** before and after immersing in Cr^3+^, Fe^3+^, and Al^3+^ aqueous solutions for 24 h. Figure S12. IR spectra of **1** before and after immersing in Cr^3+^, Fe^3+^, and Al^3+^ aqueous solutions for 24 h. Figure S13. XPS high resolution spectra of O 1*s* and N 1s of **1** before and after immersing in Cr^3+^, Fe^3+^, and Al^3+^ aqueous solutions for 24 h. Figure S14. UV-vis spectra of **1** before and after addition of Cr^3+^, Fe^3+^, and Al^3+^.

## Sample Availability

Samples of the compounds NI-mbpy-34 and **1** are available from the authors.
