# Supplementary material for: A Cd(II) Luminescent Coordination Grid as a Multiresponsive Fluorescence Sensor for Cr(VI) Oxyanions and Cr(III), Fe(III), and Al(III) in Aqueous Medium
Source: Molecules. 2021 Nov 24;26(23):7103. doi: 10.3390/molecules26237103 (PMC8659225; doi:10.3390/molecules26237103)
Supplement: Supplementary file 1 [file molecules-26-07103-s001.zip › molecules-1441580-supplementary.pdf]

Supporting Information

A Cd(II) luminescent coordination grid as a multiresponsive fluorescence sensor for Cr(VI) oxyanions and Cr(III), Fe(III), and Al(III) in aqueous medium

Kuo-Shun Liao,[1] Meng-Jung Tsai,[1] Li-Jen Hsu,[1] Chih-Min Wang[2,3]* and Jing-Yun Wu[1]*

[1]Department of Applied Chemistry, National Chi Nan University, Nantou 545, Taiwan.

[2]Institute of Bioscience and Biotechnology National (Taiwan) Ocean University, Keelung 202, Taiwan.

[3]National (Taiwan) Ocean University Center of Excellence for the Oceans, Keelung 202, Taiwan.

E-mail: aasd6307@gmail.com (K.-S.L.); s97324905@mail1.ncnu.edu.tw (M.-J.T.); Dodoadam1996@gmail.com (L.-J.H.)

* Corresponding Author: cmwang@ntou.edu.tw (C.-M.W.), twcmwang@gmail.com (C.-M.W.), jyunwu@ncnu.edu.tw (J.-Y.W.)

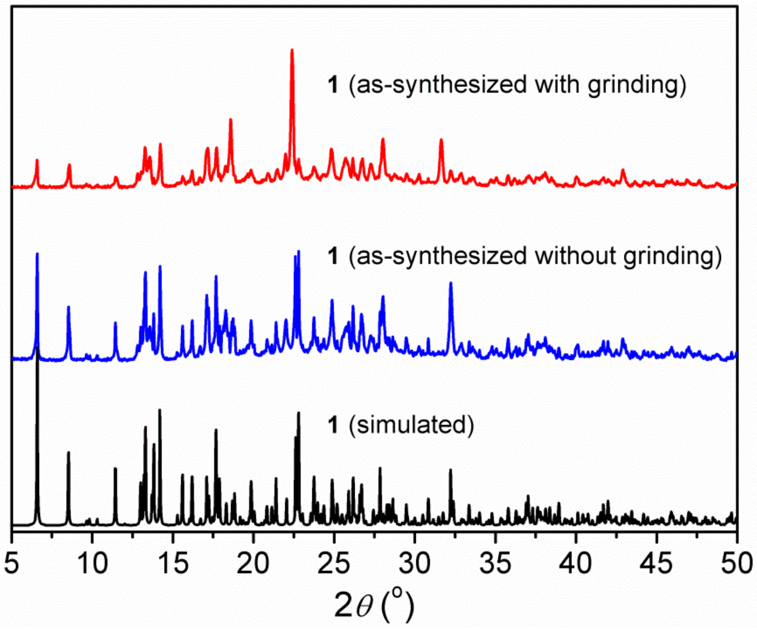

Figure S1. Simulated XRPD patterns of **1** and experimental XRPD patterns of as-synthesized **1** without and with grinding.

Table S1. Hydrogen-bonding parameters in **1**

D–H···A	*d*(D–H)	*d* (H···A)	*d* (D–A)	∠(D–H···A)	Symmetry code
O7–H101···O3	0.83	1.90	2.723(3)	171	1–x,1–y,1–z
O7–H102···O6	0.83	2.39	2.932(3)	124	–x,1–y,1–z
O8–H103···O9	0.86	2.24	3.035(5)	153	
O8–H104···O2	0.86	1.99	2.839(5)	167	1–x,1–y, –z
O9–H105···O4	0.84	2.04	2.855(4)	162	1–x,1–y,1–z

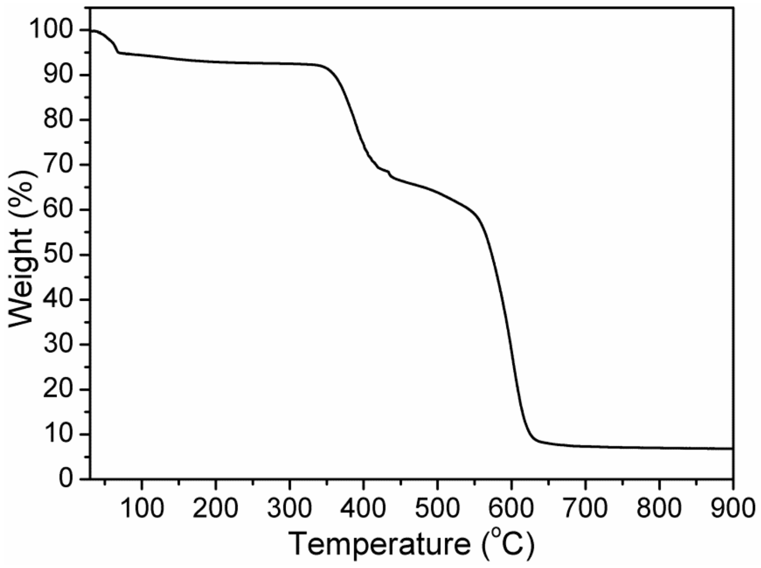

Figure S2. TG curve of **1**.

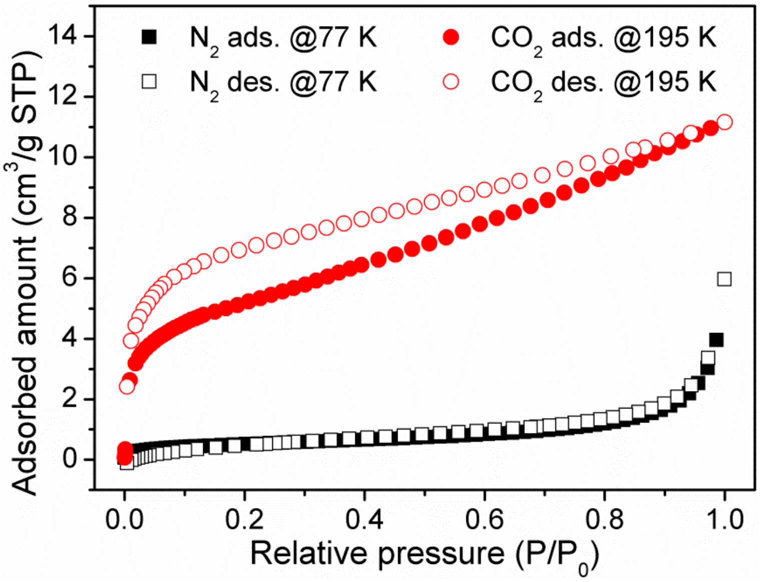

Figure S3. Gas adsorption isotherms for thermally activated **1** conducted at 77 K for N_2 and 195 K for CO_2.

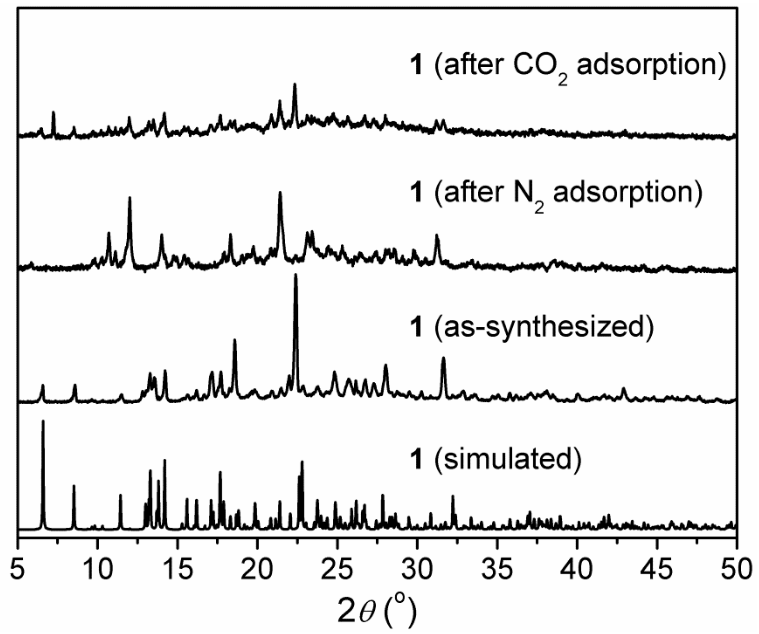

Figure S4. XRPD patterns of **1**: simulated, as-synthesized, and after N_2 and CO_2 adsorption.

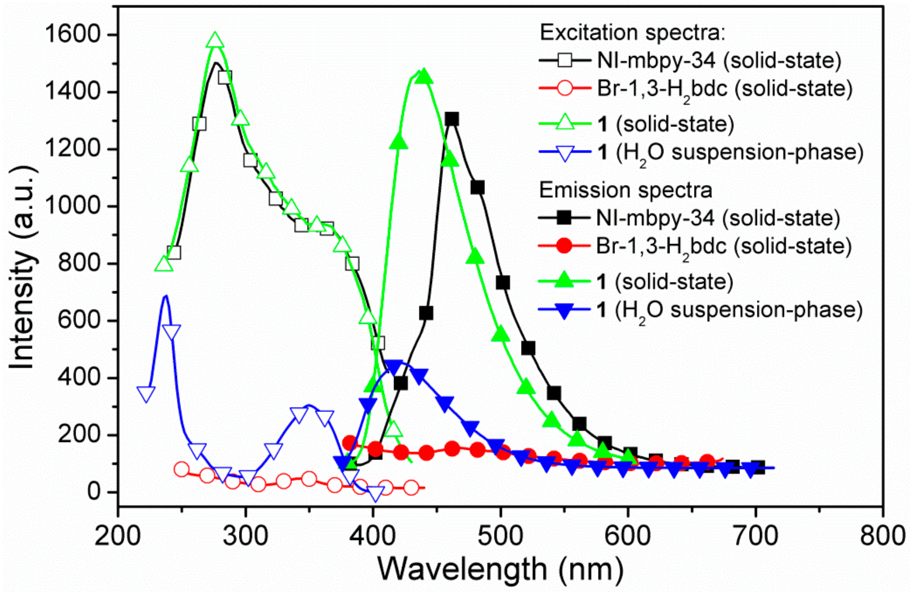

Figure S5. Fluorescence excitation and emission spectra of NI-mbpy-34, Br-1,3-H_2bdc, and **1** in solid-state as well as **1** in H_2O suspension-phase at room temperature.

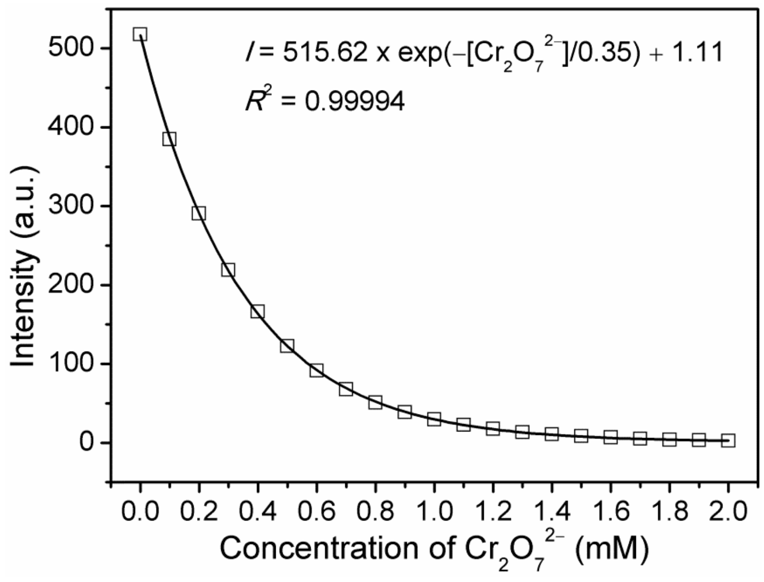

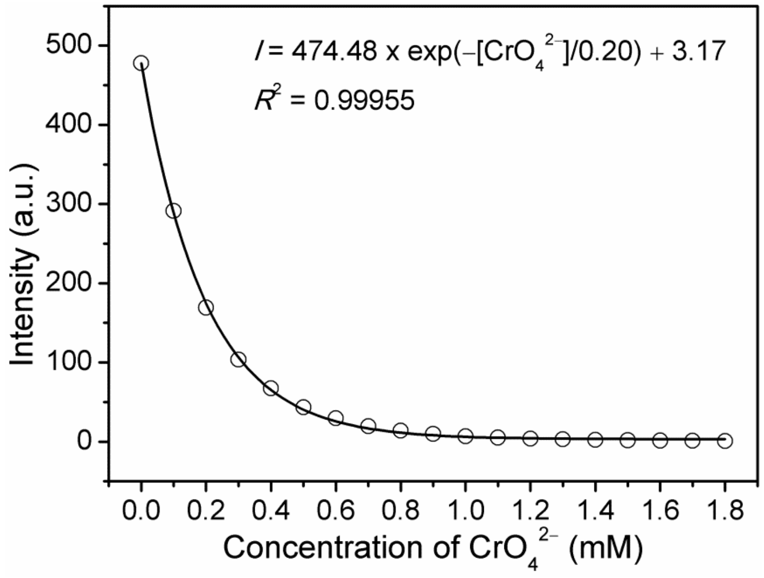

Figure S6. Fluorescence intensity traces for the H_2O suspensions of **1** upon incremental addition of $Cr_2O_7^{2-}$ and CrO_4^{2-} ions, following the first-order exponential decay. Conditions: λ_{em} = 414 nm (λ_{ex} = 365 nm).

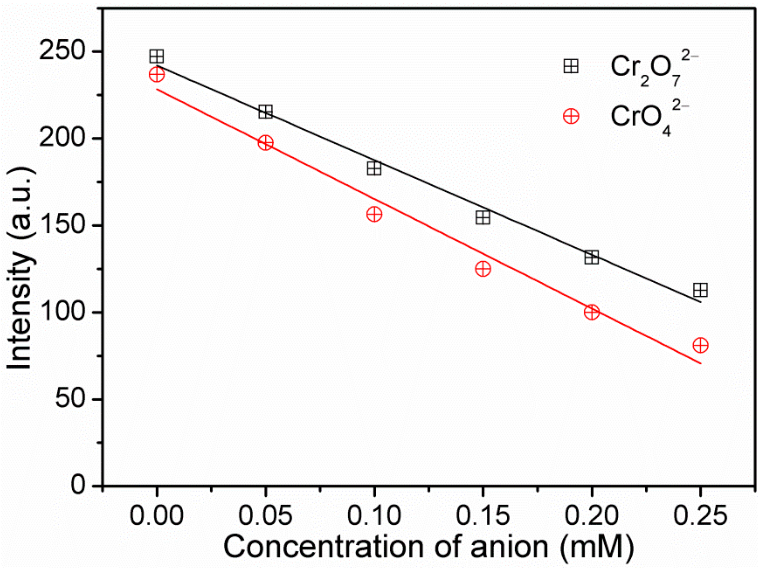

**Blank readings**	$Cr_2O_7^{2-}$	CrO_4^{2-}
#1	243.5	233.1
#2	245.8	233.6
#3	246.2	235.6
#4	247.8	238.7
#5	250.8	240.4
**Standard deviation (σ)**	**2.70**	**3.19**
**∣ Slope (m) ∣**	**544.1**	**630.7**
R^2	0.9871	0.97603
**LOD (3σ/m),** μM (ppm)	**14.91 (3.22)**	**15.15 (1.76)**

Figure S7. Linear region of fluorescence intensity for the H_2O suspensions of **1** upon incremental addition of $Cr_2O_7^{2-}$ and CrO_4^{2-} ions. The following table lists the relevant parameters of LOD for the H_2O suspensions of **1** toward $Cr_2O_7^{2-}$ and CrO_4^{2-} ions. Conditions: λ_{em} = 420 nm (λ_{ex} = 365 nm).

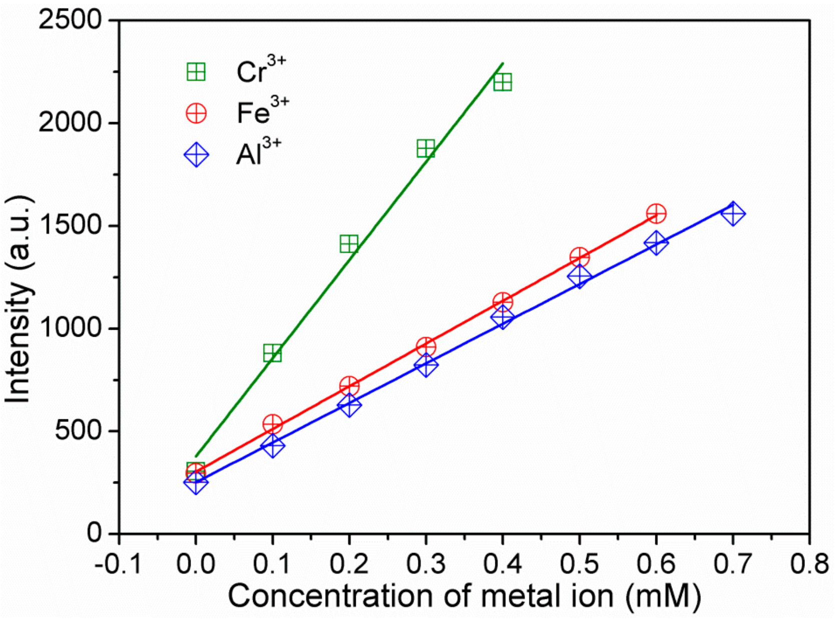

**Blank readings**	Cr^{3+}	Fe^{3+}	Al^{3+}
#1	293.3	290.4	251.6
#2	297.3	296.6	253.0
#3	298.5	290.6	256.2
#4	300.6	294.5	255.8
#5	305.5	293.7	256.4
**Standard deviation (σ)**	**4.48**	**2.65**	**2.17**
**$\lvert$ Slope (k) $\rvert$**	**4785.8**	**2080.9**	**1930.0**
R^2	0.98603	0.99898	0.99646
**LOD ($3\sigma/k$),** μM (ppb)	**2.81 (146.2)**	**3.82 (198.7)**	**3.37 (90.9)**

Figure S8. Linear region of fluorescence intensity for the H_2O suspensions of **1** upon incremental addition of Cr^{3+}, Fe^{3+}, and Al^{3+} ions. The following table lists the relevant parameters of LOD for the H_2O suspensions of **1** toward Cr^{3+}, Fe^{3+}, and Al^{3+} ions. Conditions: λ_{em} = 414 nm (λ_{ex} = 365 nm).

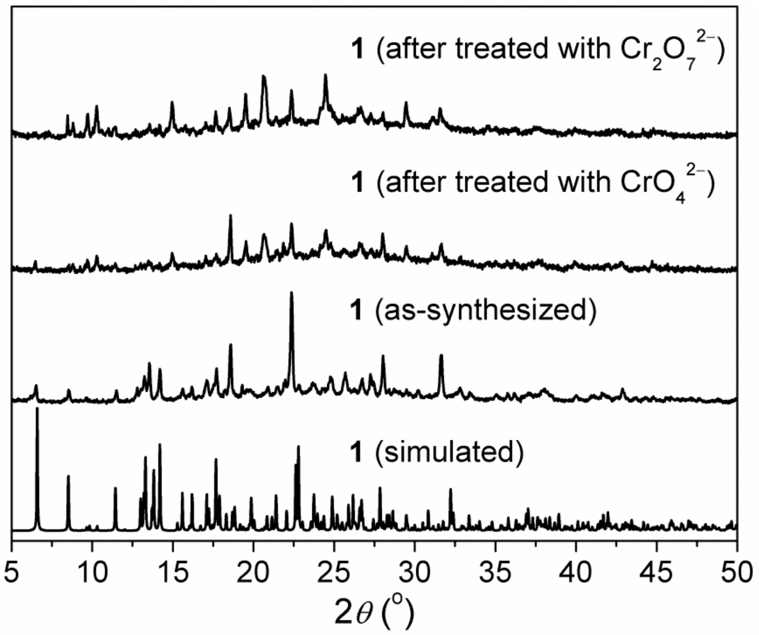

Figure S9. XRPD patterns of **1** before and after immersing in $Cr_2O_7^{2-}$ and CrO_4^{2-} aqueous solutions for 24 h.

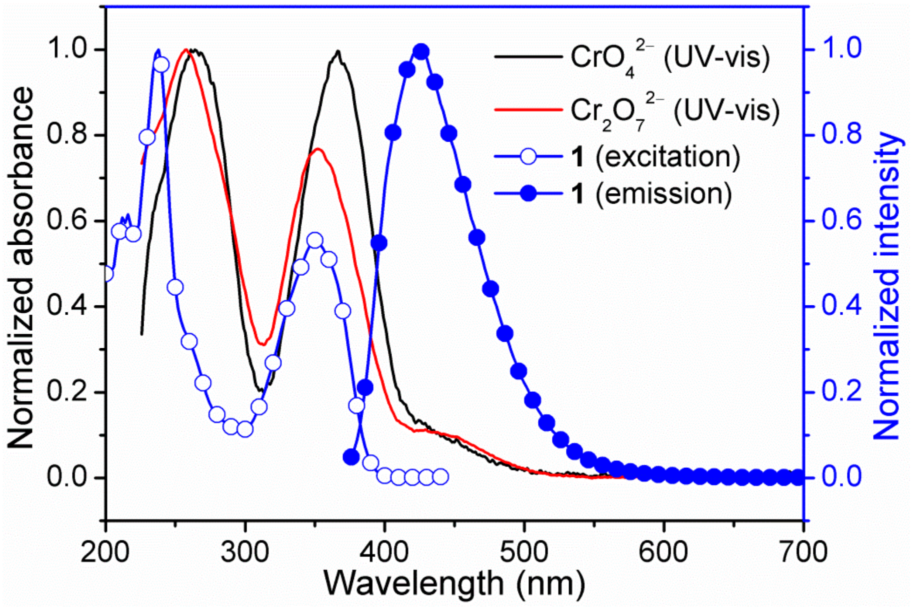

Figure S10. Spectral overlap between the normalized absorption spectra of $Cr_2O_7^{2-}$ and CrO_4^{2-} in aqueous solutions and the normalized excitation and emission spectra of **1** in H_2O suspensions.

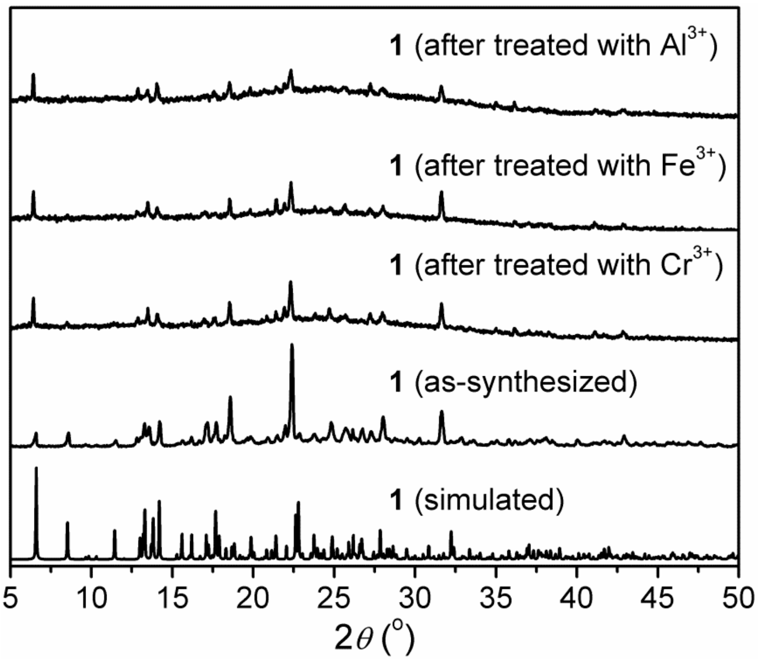

Figure S11. XRPD patterns of **1** before and after treated with Cr^{3+}, Fe^{3+}, and Al^{3+} in water for 24 h.

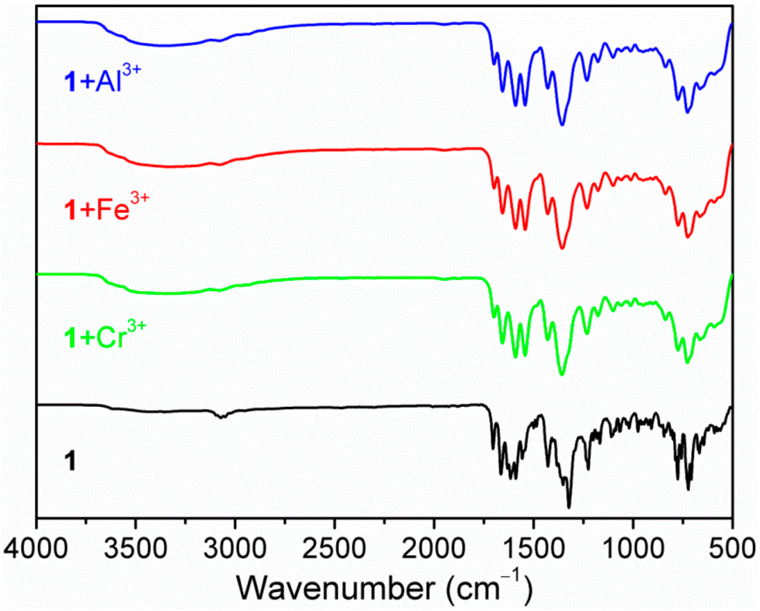

Figure S12. IR spectra of **1** before and after treated with Cr^{3+}, Fe^{3+}, and Al^{3+} in water for 24 h.

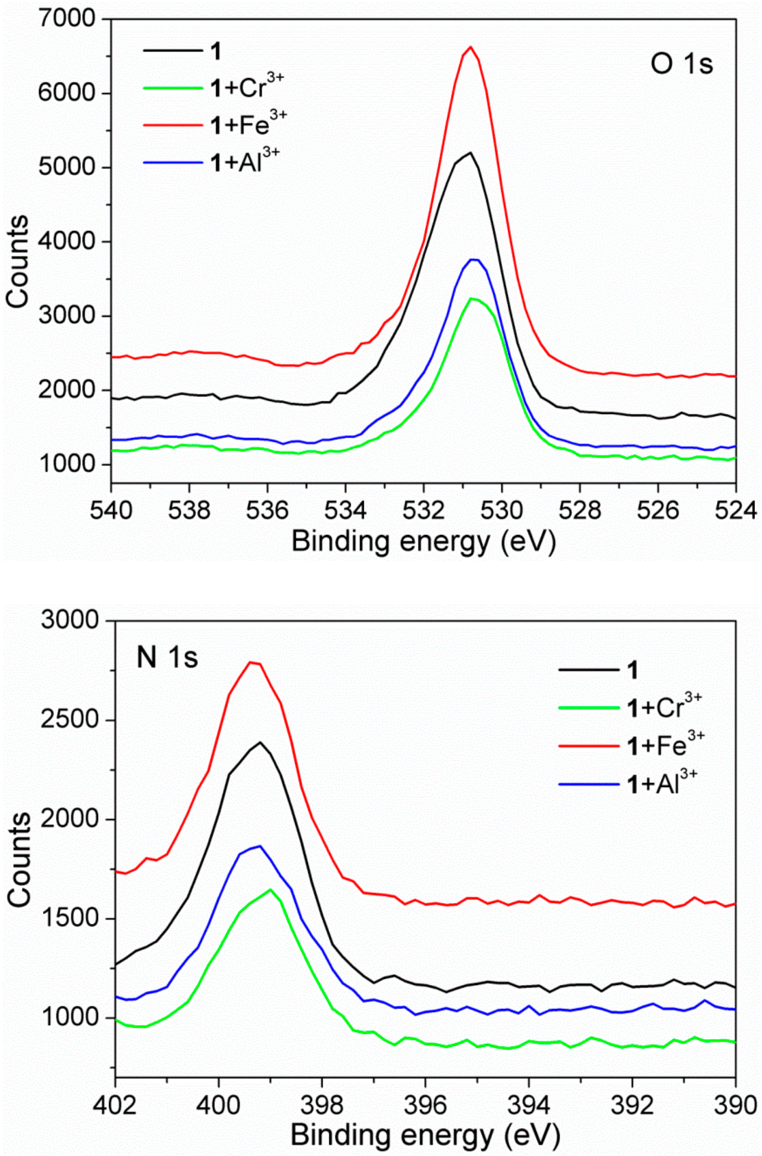

Figure S13. XPS high resolution spectra of O 1*s* and N 1s of **1** before and after treated with Cr^{3+}, Fe^{3+}, and Al^{3+} in water for 24 h.

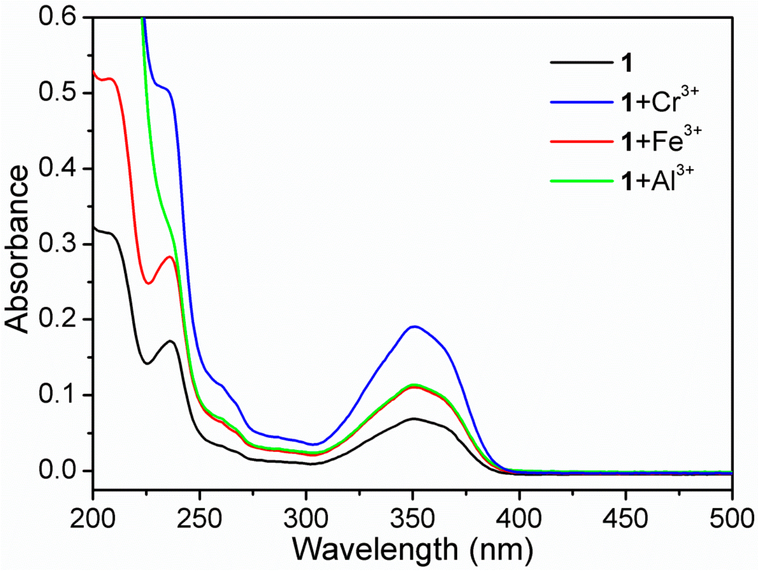

Figure S14. UV-vis spectra of **1** before and after addition of Cr^{3+}, Fe^{3+}, and Al^{3+}.
